# Long-Read Sequencing Revealed Extragenic and Intragenic Duplications of Exons 56–61 in *DMD* in an Asymptomatic Male and a DMD Patient

**DOI:** 10.3389/fgene.2022.878806

**Published:** 2022-05-09

**Authors:** Ying Bai, Ju Liu, Jinghan Xu, Yue Sun, Jingjing Li, Yong Gao, Lina Liu, Cangcang Jia, Xiangdong Kong, Li Wang

**Affiliations:** ^1^ Genetic and Prenatal Diagnosis Center, Department of Obstetrics and Gynecology, First Affiliated Hospital of Zhengzhou University, Zhengzhou, China; ^2^ Department of Neurology, First Affiliated Hospital of Zhengzhou University, Zhengzhou, China; ^3^ GrandOmics Biosciences, Beijing, China

**Keywords:** Duchenne muscular dystrophy, breakpoint analysis, *DMD* duplication, long-read sequencing, whole-exome sequencing

## Abstract

Expanded carrier screening (ECS) has become an increasingly common technique to assess the genetic risks of individuals in the prenatal or preconception period. Unexpected variants unrelated to referral are being increasingly detected in asymptomatic individuals through ECS. In this study, we reported an asymptomatic male with duplication of exons 56–61 in the *DMD* gene through ECS using whole-exome sequencing (WES), which was also detected in a male patient diagnosed with typical Duchenne muscular dystrophy (DMD). Breakpoint analysis was then performed to explore the potential mechanisms of phenotypic differences using long-read sequencing (LRS), PacBio single-molecule real-time (PacBio SMRT) target sequencing, and Sanger sequencing. Complex structural variations (SVs) on chromosome X were identified in the asymptomatic male, which revealed that the duplication occurred outside the *DMD* gene; whereas, the duplication in the patient with DMD was a tandem repeat. The phenotypic differences between the two men could be explained by the different breakpoint junctions. To the best of our knowledge, this is the first report of a breakpoint analysis of *DMD* duplication in two men with different phenotypes. Breakpoint analysis is necessary when the clinical phenotypes are inconsistent with genotypes, and it applies to prenatal testing.

## Introduction

Carrier screening determines the likelihood of individuals passing on an autosomal or an X-linked condition to their offspring. Current technological advances enable the multi-disease, pan-ethnic carrier screening, also called “expanded carrier screening (ECS),” which is performed by genotyping, sequencing, deletion/duplication analysis, or a combination of these methodologies (Kaback, 2000; Kraft et al., 2019). Since the number of copy number variation (CNV) analyses in carrier screening, pre-symptomatic, and prenatal testing has increased, more CNVs unrelated to referral are expected to be incidentally found. However, the pathogenicity of CNVs should be interpreted with caution.

Duchenne muscular dystrophy (DMD, OMIM #310200), an X-linked recessive disorder, characterized by progressive muscle degeneration and weakness, is caused by variations in the *DMD* gene (OMIM #300377), including deletions (60%–70%), duplications (10%), small rearrangements, and point mutations (Tuffery-Giraud et al., 2009). In large-scale CNV analyses performed for genetic screening in pre-symptomatic and prenatal testing, a large proportion of *DMD* variants are CNVs; thus, more CNVs in *DMD* genes unrelated to referral are expected to be incidentally found. However, it is challenging to define an explicit relationship between *DMD* CNVs (especially duplications) and the phenotypic spectrum of male carriers. Generally, to assess the potential pathogenicity of CNVs, different databases (Leiden Open Variation Database and UMD-DMD France Database) are consulted for genotype–phenotype correlations of *DMD* duplications (Tuffery-Giraud et al., 2009; Ling et al., 2020). For unreported CNVs, these may be predicted using the frameshift rule and family history.

Although individuals with different phenotypes may have the same duplication, their breakpoints can differ. However, the mechanisms underlying multi-exonic duplications have not been explored due to the technical limitations of conventional sequencing techniques. In this study, we identified the duplication of exons 56–61 in *DMD* of two men from two unrelated families, one with and the other without DMD. Long-read sequencing (LRS) and PacBio single-molecule real-time (PacBio SMRT) target sequencing were used to perform breakpoint analysis to ascertain the pathogenicity and explore the phenotypic differences, which could provide accurate guidance for genetic counseling and prenatal diagnosis.

## Materials and Methods

### Human Participants

This study involved two men from two unrelated Chinese families, with duplication of exons 56–61 in the *DMD* gene, which was confirmed by multiplex ligation-dependent probe amplification (MLPA). Informed consent was obtained from each participant to perform the investigation and genetic studies.

### MLPA

Genomic DNA (gDNA) was extracted from blood samples using standard procedures. MLPA was performed to detect the copy number of *SMN1* (OMIM #600354) using the SALSA P060 *SMA* Kit and *DMD* using the SALSA P034/P035 *DMD* Kit (MRC Holland, Amsterdam, the Netherlands).

### Long-Range PCR and Nested PCR

Intragenic mutation screening in *SMN1* (exon 1–8) was performed by long-range PCR (LR-PCR) and nested PCR as described previously in our laboratory (Kubo et al., 2015; Sun et al., 2020). LR-PCR and nested PCR were amplified using KOD FX Neo Polymerase (TOYOBO, Osaka, Japan) and 2× Taq PCR Mastermix (TIANGEN, Beijing, China), respectively. The PCR products were confirmed by 1.5% agarose gel electrophoresis and sequenced on the ABI 3130 Genetic Analyzer using the BigDye Terminator v3.1 Cycle Sequencing Kit (Applied Biosystems, Waltham, USA).

### Whole-Exome Sequencing

Genomic DNA was extracted and libraries were prepared using Illumina library construction and capture kits (Illumina, San Diego, USA) according to standard instructions (Li et al., 2021). 100× coverage of pair-end reads were obtained on an Illumina Novaseq 6,000 Sequencer (Illumina, San Diego, USA) and mapped to the hg19/GRCh37 human reference genome. Exonic and splice site variants with a minor allele frequency of less than 0.01 in public databases (dbSNP database, gnomAD, Exome Aggregation Consortium, and 1,000 Genomes) were selected. Copy number variants analysis was conducted by comparing the coverage of depth between the target sample and other male control samples in the same pipeline (Zhai et al., 2021).

### Long-Read Sequencing by Oxford Nanopore Technology (Nanopore LRS)

DNA was extracted from the fresh peripheral blood, electrophoresed to confirm its integrity, and measured using Nanodrop 2000 and Qubit 4.0 to confirm its quantity (Thermo Fisher, Massachusetts, USA). Nanopore LRS was performed using the SQK-LSK109 Kit with R9.4 flow cells on GridION X5 (ONT, Oxford, UK) according to the manufacturer’s instructions (Miao et al., 2018).In brief, 5 μg DNA was sheared to ∼ 5–25 kb fragments using Megaruptor (B06010002, Diagenode, Liège, Belgium) and size-selected to 10–30 kb with a BluePippin (Sage Science, Beverly, USA). After end repair and dA-tailing of DNA fragments, a SMRTbell™ library was purified and sequenced on R9.4 flowcells using GridION X5. The sequencing generated 2,418,823 base-called reads containing 43,641,144, and 812 bases with an average read length of 18,042 bp. The long reads were aligned to the GRCh37/hg19 reference genome using NGM-LR v0.1.4 (https://github.com/philres/nextgenmap-lr) with default parameters. Structural variations (SVs) were called by Sniffles (version 1.0.11) (Sedlazeck et al., 2018). Ribbon and IGV were used to visualize the alignment results.

### PacBio SMRT Target Sequencing of *DMD*

Genomic DNA was sequenced using PacBio SMRT target sequencing of the whole *DMD* gene (Grandomics, Beijing, China) according to the standard manufacturer’s conditions. To cover the *DMD* gene, DNA probes of 120 bases were designed and synthesized by Boke Biotechnologies (Boke, Beijing, China). Probes corresponding to repetitive sequences were removed using the Repeat Masker dataset during the design of the probes. The PacBio SMRT sequencing library was constructed using a Template Prep Kit (PacBio, Menlo Park, USA) according to the instructions. 3 μg genomic DNA was sheared to 1∼6 kb fragments by a g-Tube (#520079; Covaris, Bankstown, Australia) centrifugation. A SMRTbell™ library was prepared and sequenced on R9.4 flowcells using GridION X5. The SMRTlink 8.0 (PacBio) was used to remove low-quality reads and adapters resulting from raw sequencing data. Circular Consensus Sequence (CCS) reads were generated using the PacBio SMRT analysis software, and barcode splitting was performed by Lima. We used PBMarkDUP (PacBio) to remove potential copies in CCS reads and PBMM2 (https://github.com/PacificBiosciences/pbmm2) for comparing CCS reads to the reference genome hg19. SVs were detected using by PBSV (V9.0, https://www.pacb.com/support/software-downloads/). SVs were annotated by Annovar (http://nar.oxfordjournals.org/content/38/16/e164).

### PCR Amplification of the Junction Fragment and Sanger Sequencing

The breakpoints of the *DMD* gene identified by LRS were confirmed by PCR and Sanger sequencing. Junction fragments were amplified from gDNA using 2× Taq PCR Mastermix (TIANGEN, Beijing, China). The primers used for sequencing were designed using Gene Tool and listed in Supplementary Table S4. The breakpoint sequences were confirmed by Sanger sequencing and aligned to the reference genome hg19 using the BLAT tool (UCSC).

### Breakpoint Flanking Sequence Analysis

We manually analyzed the flanking sequence of each breakpoint and identified microhomology. We selected 100bp reads upstream and downstream of each breakpoint to search for repetitive elements using the “Repeat Masker” program of the UCSC Genome Browser.

## Results

### Clinical Description and Genetic Analysis

**FIGURE 1:**
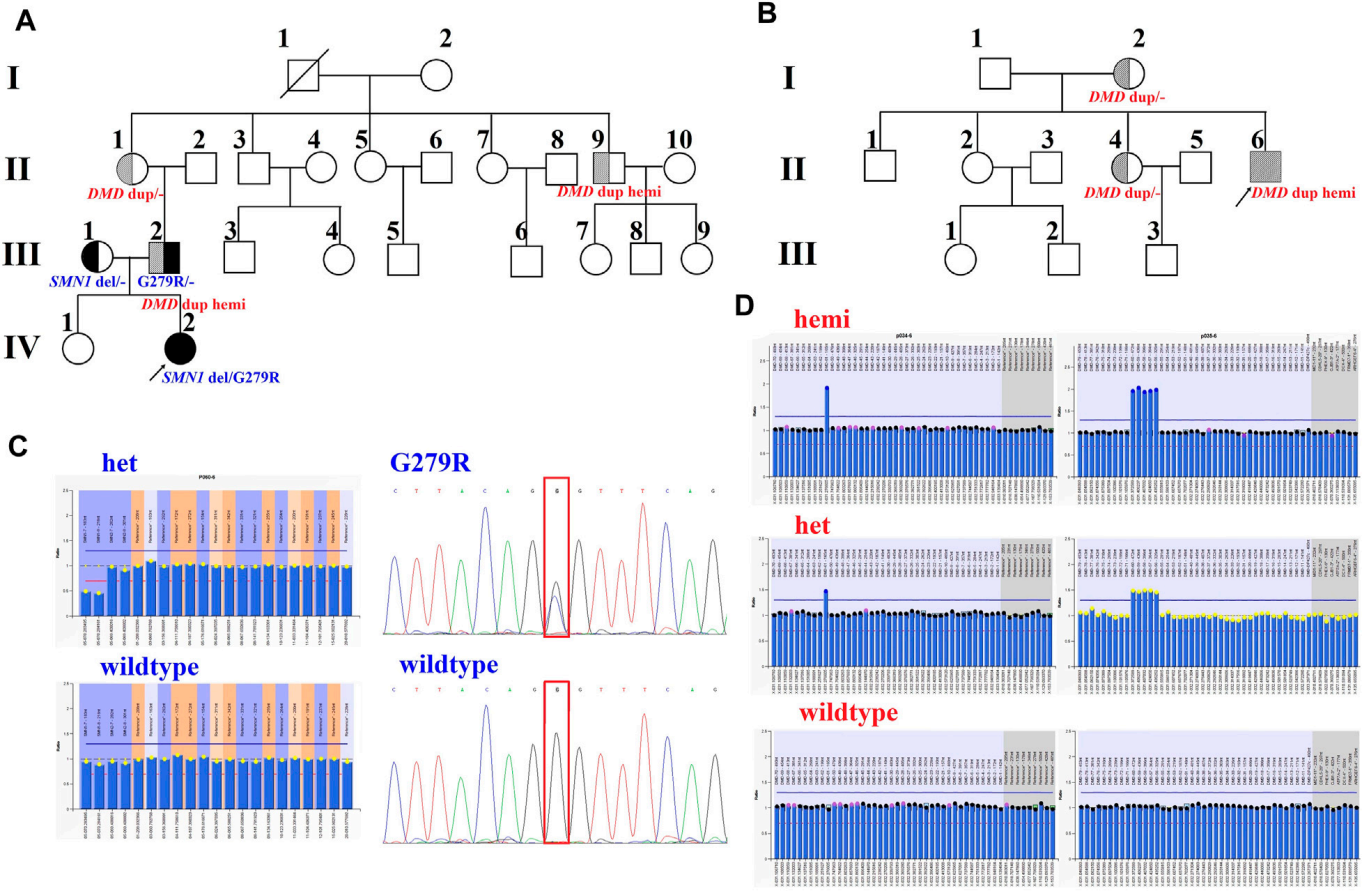
Pedigrees and genotypes of two families with the duplication of exons 56–61 in *DMD*. **(A)** Pedigrees of family 1 showed segregation of the *SMN1* mutations. Moreover, the male (III:2) and his uncle (II:9), with the duplication of exons 56–61 in *DMD*, had no symptoms of DMD. **(B)** Pedigrees of family 2 showed segregation of *DMD* duplication. **(C)** MLPA analysis and LR-PCR results showed the proband (IV:2) with compound heterozygous mutations (exons 7–8 deletion and p. G279R) in *SMN1* inherited from her parents. **(D)** Duplication of exons 56–61 in *DMD* was confirmed by MLPA analysis in two families.

Family 1 was a non-consanguineous couple that visited our hospital because they gave birth to a female patient (IV:2) diagnosed with spinal muscular atrophy (SMA1; OMIM #253300) (Figure 1A). Molecular genetic testing of SMA in the IV:2 female and ECS in the couple (III:1 and the III:2) were performed simultaneously in order to reduce the risk of having an affected child associated with genetic disease. Compound heterozygous mutations, exons 7–8 deletion, and c.835G > C p(G279R) in the survival motor neuron (*SMN1*) gene were detected in the IV:2 female, which were inherited from her parents (Figure 1C, Supplementary Figure S1, S2). The quality control of the WESdata is summarized in Supplementary Table S1. Furthermore, no other pathogenic variant was found in the couple by single-nucleotide variants and indel analysis of whole-exome sequencing (WES). However, duplication of exons 56–61 in the *DMD* gene was found in the Ⅲ:2 male (Figure 1A) by WES-based CNV analysis (Supplementary Figure S3), which was confirmed by MLPA (Figure 1D). Since he was asymptomatic and there was no family history of DMD, other members of family 1 were tested (Figure 1A, Ⅱ:1, Ⅱ:3, and Ⅱ:9). MLPA analysis identified the same duplication in the mother (Ⅱ:1) and uncle (Ⅱ:9) of the male (Figure 1D). The man (Ⅲ:2) and his uncle (Ⅱ:9) were 33 and 44 years old, respectively; they had slightly elevated serum creatine kinase (CK) concentration (288 IU/L and 204 IU/L, reference range: 24–195 IU/L) and normal surface electromyography of the flexor and extensor muscles (EMG).

In family 2, the 23-year-old man (Ⅱ:6) was presented with a typical DMD (Figure 1B). He was unable to run or jump and became wheelchair dependent at the age of eleven. His CK concentration was 12,696 IU/L, which was 60 times higher than the upper normal limit. He had been dependent on 24 h ventilation for 7 years before the visit. The duplication of exons 56–61 in *DMD* was identified in the Ⅱ:6 male patient by MLPA analysis, which was inherited from his mother.

### Breakpoint Analysis

**FIGURE 2:**
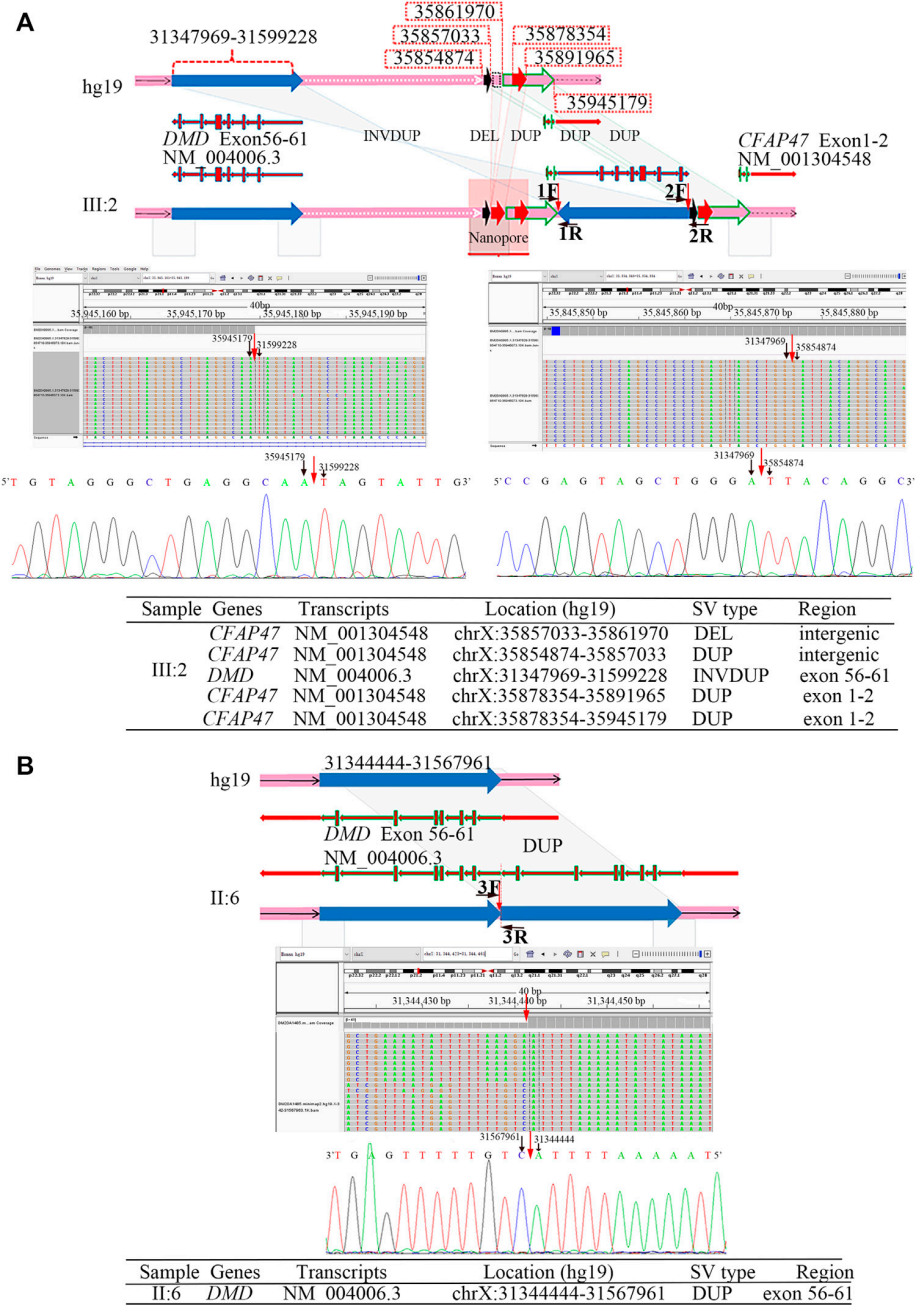
Breakpoint analysis in two men from unrelated families with the duplication of exons 56–61 in *DMD*. **(A)** Complex SVs on chromosome X were identified in the asymptomatic male (family 1, Ⅲ:2) by LRS, target PacBio SMRT sequencing, and Sanger sequencing. Schematic representation of complex SVs revealed a duplication–deletion–inversion duplication–duplication–duplication was outside the *DMD* gene. An integrative genomics viewer (IGV) screenshot of target PacBio SMRT sequencing showed that 5′ and 3′ breakpoints of inversion duplication were precisely located at chrX:35945179 and chrX:35854874, which was confirmed by Sanger sequencing. The breakpoints’ sequences of INVDUP are shown in the 5′-3′ orientation. **(B)** A tandem repeat of exons 56–61 in the *DMD* gene was identified in the male (Ⅱ:6) from family 2 by the target PacBio SMRT sequencing and Sanger sequencing. A schematic representation showed the junction of breakpoint was located at chrX:31567959, which was confirmed by Sanger sequencing. The breakpoint sequences of a tandem repeat are shown in the 3′-5′ orientation.

The results of Nanopore LRS and PacBio SMRT target sequencing in the male of family 1 revealed complex structural variations (SVs) on chromosome X, comprising a 251.4 kb inversion duplication (INVDUP, *DMD* gene); three duplications of 2.3, 13.5, and 66.6 kb (*CFAP47* gene); and a 4.9 kb deletion. Sanger sequencing revealed that the 5′ and 3’ breakpoints of INVDUP were located at chrX:35945179 and chrX:35854874, respectively, which indicated that the duplication occurred outside the *DMD* gene (Table 1; Figure 2; Supplementary Figure S4–7; Supplementary Table S2-3). In the patient of family 2, PacBio SMRT target and Sanger sequencing confirmed that the duplication was a tandem repeat and that the junction of breakpoint was located at chrX:31567961 (Table 1; Figure 2; Supplementary Figure S4–7).

**TABLE 1:** Molecular signatures at breakpoint sequence.

Family	Case	Breakpoint (chrX)	Confirmation methods	Repeated elements			
				SINE	LINE	LTR	Satellite
F1	III:2	31347969Δ	Sanger, LRS, SMRT target sequencing	AluSc	—	—	—
	III:2	31599228*	Sanger, LRS, SMRT target sequencing	—	—	—	—
	III:2	35854874Δ	Sanger, LRS, SMRT target sequencing	AluSp	—	—	—
	III:2	35857033	LRS, SMRT target sequencing	—	—	MER83B-int	—
	III:2	35861970	LRS, SMRT target sequencing	—	L1MC1	—	—
	III:2	35878354	LRS, SMRT target sequencing	AluSg	L1MA6	—	—
	III:2	35891965	LRS, SMRT target sequencing	AluSx1	L1MB3	—	—
	III:2	35945179*	Sanger, LRS, SMRT target sequencing	AluJb	—	—	—
F2	II:6	31344444#	Sanger, SMRT target sequencing	—	—	—	AT_rich
	II:6	31567961#	Sanger, SMRT target sequencing	—	—	—	—

Repeated elements were defined according to Repeat Masker. SINE: short interspersed nuclear element, LINE: long interspersed nuclear element, LTR: long terminal repeat elements, low complexity: low-complexity repeats. *, confirmed by Primer F1R1; Δ, confirmed by Primer F2R2; #, confirmed by Primer F3R3.

To determine the mechanism by which SVs were formed, the mutational signatures of the breakpoint junctions were investigated. The microhomology analysis performed between the paired flanking sequences identified 4 bp microhomology “TTGC” and 27 bp microhomology “CTG​CCT​CAG​CCT​CCC​GAG​TAG​CTG​GGA” were identified in the junctions of INVDUP and 2 bp microhomology “AT” in the junction of tandem repeat (Supplementary Figure S5). To obtain sufficient information, we used 100 bp flanking reads and performed the motif analysis. We found that these segments around the breakpoints are considered to be derived from repeating elements overlapped with short interspersed nuclear elements (SINEs, Alu family) and long interspersed nuclear elements (LINEs, L1 family) in the asymptomatic male (Ⅲ:2, family 1) and low-complexity repeats (AT rich) in the Ⅱ:6 male from family 2 (Table 1, Supplementary Figure S8).

## Discussion

In this study, we identified the same duplication of exons 56–61 in the *DMD* gene of two men with different phenotypes. Moreover, we explored, for the first time, the potential mechanism for phenotypic differences through breakpoint analysis. *DMD* duplication as a tandem repeat resulted in a frameshift variation that could produce a pathogenic C-terminally truncated protein p.(Thr3055Argfs*5). The breakpoints of *DMD* duplications occurred mainly in the introns, which are too large to be detected by MLPA or WES analysis (Ling et al., 2020). LRS is an alternative approach to analyzing SVs. Sequencing results revealed that breakpoints of *DMD* duplications were located in different genomic regions, which were consistent with the predictions from PacBio SMRT target sequencing data. The phenotypic differences between the two males could be explained by the differences in the breakpoint junctions.

Several studies have identified *DMD* duplications in females or fetuses during carrier screening and pre-symptomatic and prenatal testing, which were not detected in any male family member. For example, the duplication of exons 3–7 in *DMD* was incidentally identified by array comparative genomic hybridization in a girl without a family history of DMD, who was affected by cystic fibrosis and maturity-onset diabetes of the young type 5 (MODY5) due to a classical 17q12 microdeletion (Nguyen et al., 2015). Moreover, an out-of-frame duplication of exons 51–62 in *DMD* was detected in a female fetus based on the data of non-invasive prenatal testing (NIPS), which was inherited from the mother and not present in any male family member (Brison et al., 2019). Since the family segregation was not informative, the variant classification of *DMD* duplication was a major challenge. Therefore, breakpoint mapping is recommended to ascertain the pathogenicity, and it could provide accurate guidance for genetic counseling and prenatal diagnosis.

Several different mechanisms have been hypothesized to initiate genomic recombination: 1) homologous recombination including non-allelic homologous recombination (NAHR), gene conversion, single-strand annealing, and break-induced replication; 2) non-homologous end joining (NHEJ); 3) microhomology-mediated replication-dependent recombination (MMRDR); 4) LINE-1-mediated retrotransposition; and 5) telomere healing (Chen et al., 2010). Among these, MMRDR and NHEJ are the two main mechanisms involved in *DMD* intragenic deletions in the previous studies (Ling et al., 2020). In order to yield insights into the mechanism of SV formation, mutational features around breakpoint junctions were analyzed. In the present study, microhomologies were found in both *DMD* duplication individuals. These findings are consistent with the hypothesis that MMRDR might lead to replication fork stalling and template switching, which could produce complex deletions and duplication rearrangements (Ling et al., 2020). We also found SINE (Alu) and LINE (L1) elements near the breakpoint junctions in the SVs of the asymptomatic male, which was consistent with other studies (Gu et al., 2016). Repetitive sequences (Alu, LINE, and human endogenous retroviral element repeats) may form aberrant secondary structures, which are particularly susceptible to rearrangement. Repetitive elements have been recognized as SV hotspots and may give rise to recurrent SVs mediated by NAHR (Weckselblatt and Rudd, 2015). Therefore, we predicted that these repeat regions might be fragile and prone to chromosomal abnormalities caused by interference with DNA replication, recombination, and repair (Sen et al., 2006). Other junctions’ sequence of breakpoints outside the *DMD* gene need to be further validated by Sanger sequencing.

## Conclusion

To the best of our knowledge, this is the first report of breakpoint analysis in two men with different phenotypes carrying the same *DMD* duplication. Therefore, caution is recommended in the use of carrier screening duplications for clinical prediction, especially with no family history of genetic disease. The breakpoint analysis is necessary when clinical phenotypes are inconsistent with genotypes, which also applies to prenatal testing. Our results reinforce the importance of detailed clinical evaluation, precise molecular detection, and appropriate genetic counseling.

## Data Availability

The datasets for this article are not publicly available due to concerns regarding participant/patient anonymity. Requests to access the datasets should be directed to the corresponding authors.
