# Supplementary material for: Long-Read Sequencing Revealed Extragenic and Intragenic Duplications of Exons 56–61 in DMD in an Asymptomatic Male and a DMD Patient
Source: Front Genet. 2022 May 9;13:878806. doi: 10.3389/fgene.2022.878806 (PMC9125615; doi:10.3389/fgene.2022.878806)
Supplement: Supplementary file 1 [file DataSheet1.docx]

**SUPPLEMENTARY MATERIALS**

**Supplementary Figure 1. Screenshot of the exome alignment of c.835G>C p.(G279R) in the parents of family 1 (the red box).**


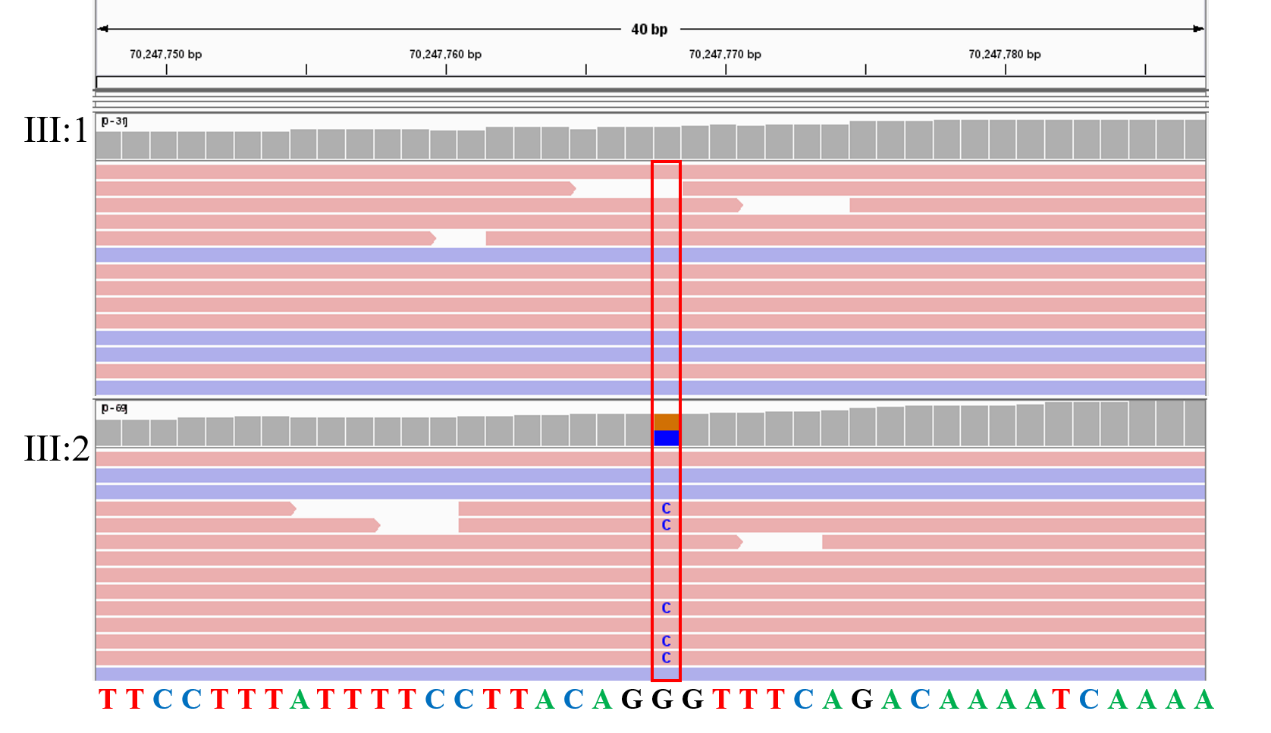
 Red: sense strand, blue: antisense strand.

**Supplementary Figure 2. The copy number of *SMN1* exon 7 in the parents of family 1 based on WES.**


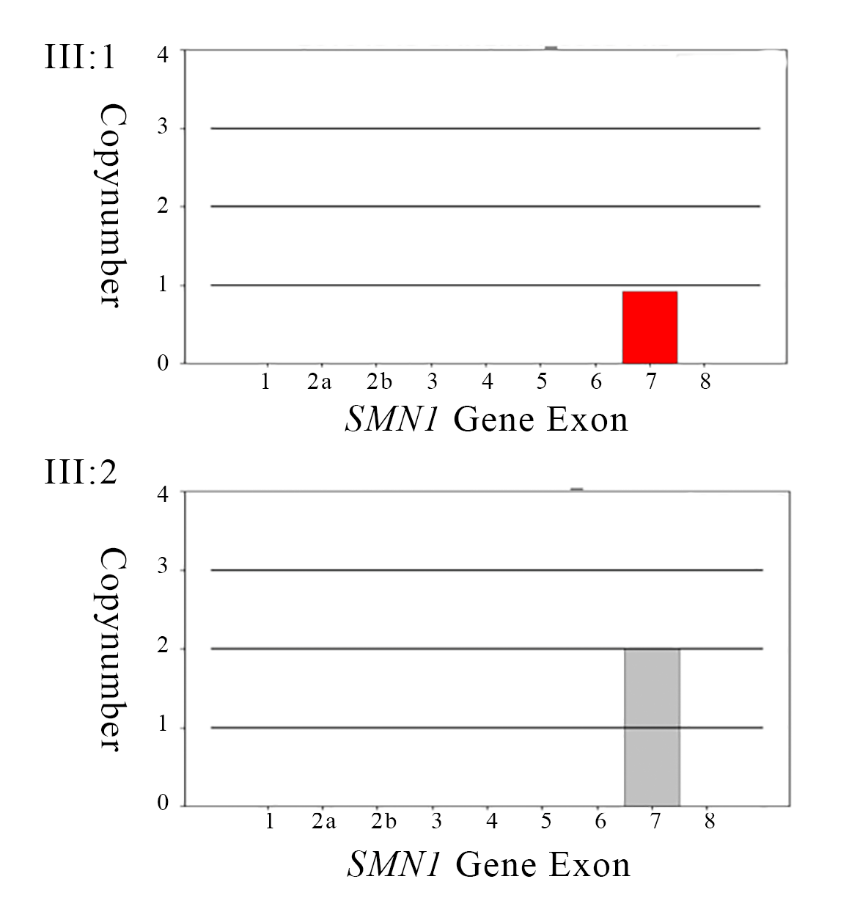


**Supplementary Figure 3. The copy number of *DMD* gene in the parents of family 1 based on WES.**


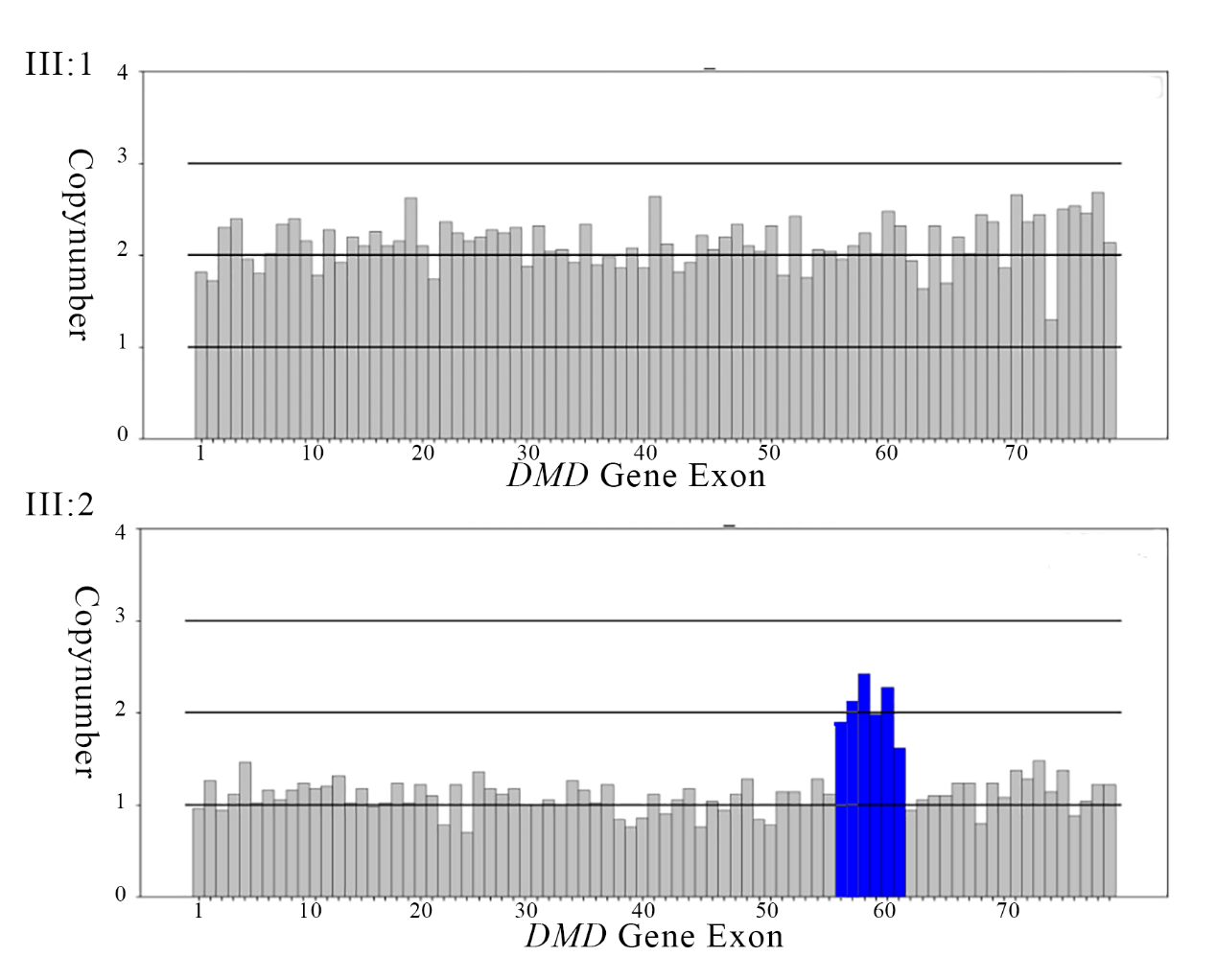


**Supplementary Figure 4.** **The PCR results of three breakpoints by agarose gel electrophoresis.**


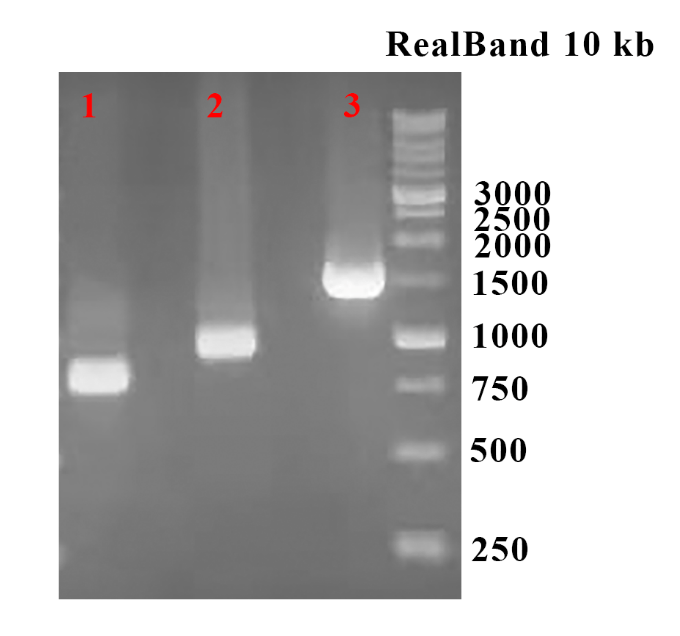


1. Primer pair 1F/1R amplified in Ⅲ:2 of family 1; 2. Primer pair 2F/2R amplified in Ⅲ:2 of family 1; 3. Primer pair 3F/3R amplified in Ⅱ:6 of family. The PCR products were 843 bp, 1291 bp and 1458bp, respectively. While those primer pairs cannot be amplified in normal man.

**Supplementary Figure 5. Junction sequences in two families aligned to the corresponding regions of the reference sequences.**

actgtagtcccagctacttgtagggctgaggcaagaggatcacttaaacccaagaggtagaggct

actgtagtcccagctacttgtagggctgaggcaatagtattgctaaaataaaggagaaggcaactt

tcccataaagtcccagcttgattttatatgctgcaatagtattgctaaaataaaggagaaggcaactt

35945179

*CFAP47*

Breakpoint 1

31599228

*DMD IN61*

cccaggttcaagcgattctcctgcctcagcctcccgagtagctgggactataggtgcgcgccacctc

cccaggttcaagcgattctcctgcctcagcctcccgagtagctgggattacaggcatgtgccacctt

Breakpoint 2

cccaggttcaagcgattcttctgcctcagcctcccgagtagctgggattacaggcatgtgccacctt

*CFAP47*

31347969

35854874

*DMD* IN55

cttgatatttaaaagaatcgtttatgagtttttgtcatcatgaatgtttgacaacaagattatctgtgaaca

gcttgaactaaatagctgaaaatatttttaaagaattttaaaaatattataaattctagatcaatatgaaat

cttgatatttaaaagaatcgtttatgagtttttgtcattttaaaaatattataaattctagatcaatatgaaat

31567961

31344444

*DMD IN55*

*DMD IN61*

Breakpoint 3

Regions of microhomology are enclosed in black boxes. Arrowheads indicate inferred breakpoint sites.

**Supplementary Figure 6. The overview of the breakpoint (ChrX:31347969) after analyzing the original BAM file of Ⅲ:2 of family 1.**


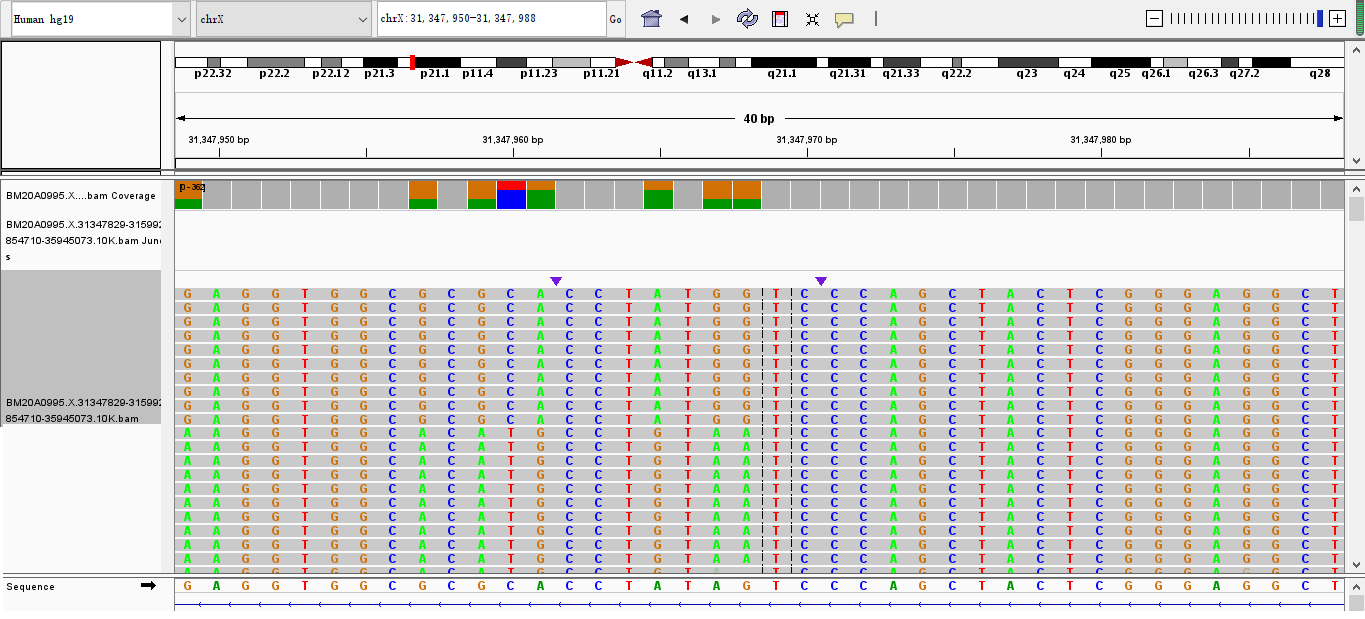


**Supplementary Figure 7. The overview of the breakpoint (ChrX:** **31599228) after analyzing the original BAM file of Ⅲ:2 of family 1.**


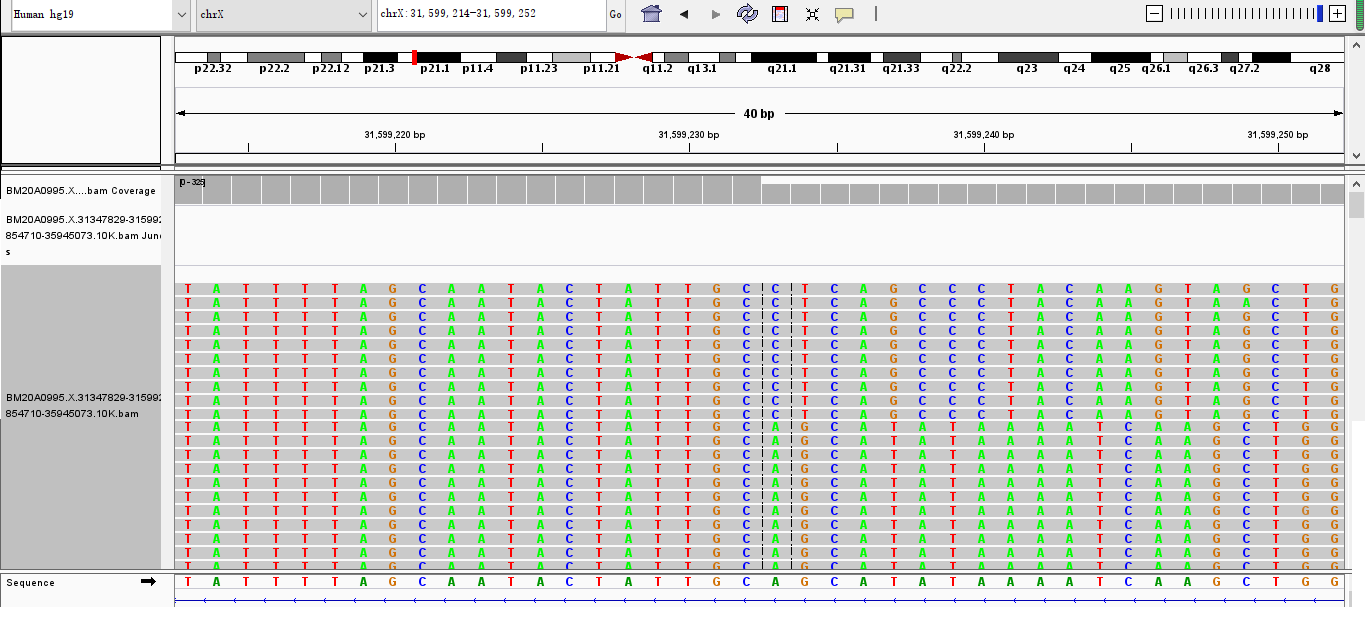


**Supplementary Figure 8. Repetitive elements detected within 100bp breakpoint regions.**

**
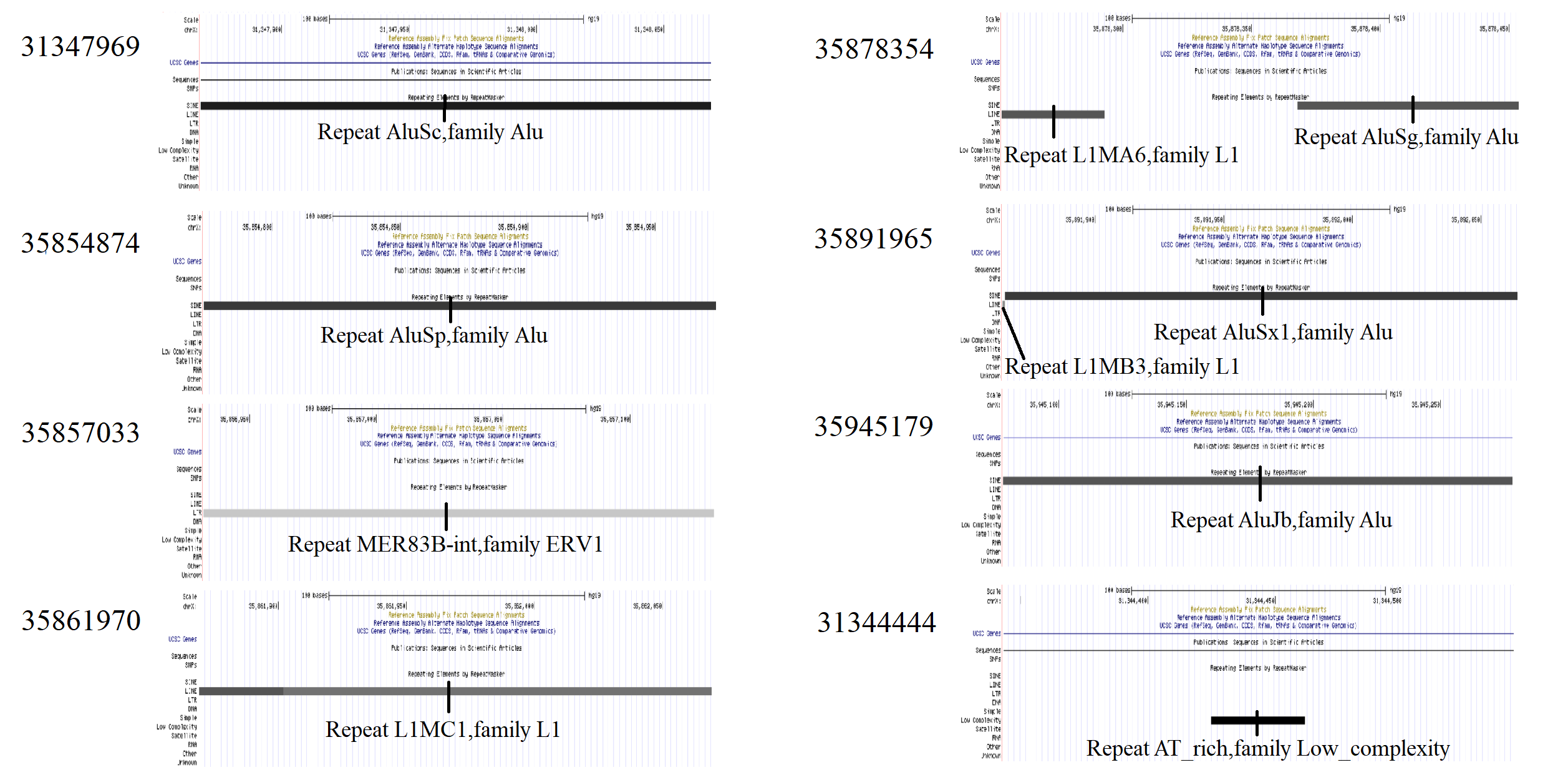
**

**Supplementary Table 1. Quality control of the WES data of the parents of family 1(Ⅲ:1 and Ⅲ:2).**

| **Sample** | **20×Coverage** | **Data size (Gb)** | **Capture efficiency (%)** | **Duplication rate (%)** | **Ave. sequencing depth (×)** |
| --- | --- | --- | --- | --- | --- |
| Ⅲ:1 | 97.98% | 8.8 | 62 | 19.19 | 104 |
| Ⅲ:2 | 97.72% | 9.2 | 61 | 19.16 | 108 |

**Supplementary Table 2. Summary of base alignments of Nanopore LRS.**

| **Sample** | **Pass bases** | **Mapped Bases** | **Pass reads** | **Mapped Reads** | **Mapping Rate** | **Mean Length** | **Depth** |
| --- | --- | --- | --- | --- | --- | --- | --- |
| Ⅲ:2 in family 1 | 43.6 G | 41.9 G | 2.4 M | 2.3 M | 95.76% | 18.0 kb | 14.55 |

Depth: Count based on Genome size (3Gbp).

**Supplementary Table 3. Summary of base alignments of PacBio SMRT target sequencing in two families.**

| Sample | Ⅲ:1 in family 1 | Ⅱ:6 in family 2 |
| --- | --- | --- |
| Pass bases | 709,347,244 | 1,007,566,599 |
| Target bases | 473,130,228 | 568,818,776 |
| Target average depth | 209.13 | 251.42 |
| Coverage bases | 2,261,784 | 2,261,973 |
| Coverage bases (20x) | 2,259,129 | 2,258,853 |
| Coverage ratio (20x) | 99.86% | 99.84% |
| Pass reads | 240,731 | 313,264 |
| Target reads | 159,790 | 176,238 |
| Mean Reads length | 2947 | 3216 |

**Supplementary Table 4. Primers for Sanger sequencing.**

| **Primer ID** | **Primer sequence** | **Location (hg19/GRCh37)** | **PCR product of mutant type** |
| --- | --- | --- | --- |
| *DMD*-Breakpoint-1F | TTGCCCACCACCTACAGAATTAA | chrX:35944883-35944905 | 843 bp |
| *DMD*-Breakpoint-1R | GGCGGTTTGGTAATGGACTGA | chrX:31598687-31598707 |  |
| *DMD*-Breakpoint-2F | CCCTTTTGTTTTCCTCCACTGTA | chrX:31348352-31348374 | 1291 bp |
| *DMD*-Breakpoint-2R | CCACTGGGGACACAAAGAGATAC | chrX:35855427-35855449 |  |
| *DMD*-Breakpoint-3F | CCCCTCCATATCTGCTCCTTTAA | chrX:31567297-31567319 | 1458 bp |
| *DMD*-Breakpoint-3R | GCTGCCCTGGAATTACACGTAGT | chrX:31345210-31345232 |  |

1:5-prime of INVDUP in family 1; 2:3-prime of INVDUP in family 1; 3: the junction of tandem repeat in family 2.
